# Sex-changing patterns of Akoya pearl oyster (*Pinctada fucata*)

**DOI:** 10.1186/s40851-018-0098-7

**Published:** 2018-06-05

**Authors:** Jeane Siswitasari Mulyana, Toshiharu Iwai, Masaharu Takahashi, Achmad Farajallah, Yusli Wardiatno, Chiemi Miura, Takeshi Miura

**Affiliations:** 1 Department of Biology, Bogor Agricultural University, Bogor, West Jawa Indonesia; 2 Laboratory of Fish Reproductive Physiology, Graduate School of Agriculture, Ehime University, Matsuyama, Ehime Japan; 3 Pearl Oyster Research Laboratory, Shimonada Fisheries Cooperative, Uwajima, Ehime Japan; 4 Department of Aquatic Resources Management, Bogor Agricultural University, Bogor, West Jawa Indonesia; 5 Department of Global Environment Studies, Faculty of Environmental Studies, Hiroshima Institute of Technology, Hiroshima, Hiroshima Japan

**Keywords:** Gene expression, Germ cells, Gonadal stage, Hermaphrodite, Male ratio

## Abstract

**Background:**

Pearl production by transplantation in Akoya pearl oyster **(***Pinctada fucata***)** is a biotechnology developed in Japan that skillfully utilizes the pearl-forming ability of oysters. In this method, cultured pearls are formed from a pearl nucleus and a small piece of mantle transplanted into the gonads of recipient pearl oysters. In this study, we hypothesized that the sex of the recipient pearl oyster might affect the quality of pearl produced. While some previous studies have examined the sex of Akoya pearl oyster, detailed information is lacking.

**Results:**

To investigate sex in Akoya pearl oyster, we collected small gonadal fragments from 1-year-old pearl oysters by biopsy. Using the collected gonad fragment, the sex of the oysters was determined by microscopic observation, and the remaining samples were stored for gene expression analyses. All oysters were labeled to distinguish each individual for serial samplings every four months over the 2-year study period. At the start of experiment, nearly all of the pearl oysters were male, but the male:female ratio ofmale decreased over the course of the experiment. Interestingly, the number of males increased after spring, during the breeding season. This suggests that, in pearl oyster, sex is affected by season. Expression analysis of sex-related genes (*Dmrt2, Vtg, Zp*) indicated that all genes were expressed in all individuals and all periods.

**Conclusions:**

These results suggest that Akoya pearl oysters are hermaphroditic, and that females appear as necessary, such as during the breeding season. These findings could contribute to higher efficiency and quality of pearl cultivation.

## Background

Cultivation of Japanese Akoya pearl oyster (*Pinctada fucata*) for pearl production is an important traditional marine industry in Japan, with a history of more than 100 years. The characteristics of cultured pearls are affected by two kinds of pearl oysters: the donor, which provides the small piece of mantle to be transplanted, and the recipient, in which the pearl nucleus and a small piece of mantle are transplanted to produce a pearl. Generally, the brightness, luster, and color of pearls are affected by the donor oyster, while the thickness of the nacre is affected by the recipient. In pearl culture, only the recipient oyster needs to have a rapid growth rate and disease-resistance; hence, the quality of produced pearls has been underestimated. In transplantation, the pearl nucleus and a small piece of mantle are transplanted into the gonads of the recipient pearl oyster, thus the gonadal condition of the recipient pearl oyster should affect the efficiency of pearl culture [1]. Since germ cells, such as sperm and egg, in the gonads are directly linked to the quality and efficiency of cultured pearls, most pearl farmers have focused on managing the condition of the gonads in pearl oysters, which is a costly and labor-intensive process.

Although the sex of pearl oysters has been studied in the past, detailed information on the sex characteristics of this specied is lacking. Understanding of the sex of the pearl oyster is very important for their breeding, as well as for pearl culture. In a previous study [2], we found that the sex of the recipient pearl oyster greatly affected the quality of cultured pearls. Male recipient oysters produced commercially valuable pearls at higher rates than female recipient oysters. The average percentage of low-quality pearls from male recipient oysters was lower than that of the female recipient oyster. Differences in pearl formation ability between male and female oyster in pearl formation can be explained by nacre growth. Nacre grew evenly every month in males, while there was a nacre growth stagnation phase between July and August and between September and October in female oysters [2]. Male Akoya pearl oyster is more favorable for pearl culture because it produces high-quality pearls on average. However, Akoya pearl oyster’s sexual cycle is complex. There are three sexes in Akoya pearl oyster: male, female, and hermaphrodite [3]. Interestingly, several studies have reported that oyster species have the ability to change sex [4–9], which adds further complexity. Achieving a better understanding of sex-changing patterns is important for Akoya pearl oyster culture, due to the differences in pearl formation ability in male and female oysters. The aim of the present study is to describe sex characteristics in Akoya pearl oyster using histology and gene expression analysis.

## Methods

### Biopsy of gonad fragment of Akoya pearl oysters

In this study, we used 256 Akoya pearl oysters (*Pinctada fucata*) produced from a single pearl oyster hatchery (Pearl Oyster Research Laboratory, Shimonada Fisheries Cooperative, Ehime Prefecture, Japan) in February and March 2013. All oysters were marked to distinguish each individual for serial sampling. A fragment of gonad of Akoya pearl oyster was taken by biopsy method once in every four months from April 2014 until August 2016, for a total of seven sampling periods. Biopsy method was performed by needle aspiration through the gonad after the oysters were anesthetized with 0.35 M MgCl_2_ [10]. Approximately 100 mg gonad fragment was taken from each individual. Each sample was then divided into two parts. One part was fixed in Davidson solution and used for smeared-slide glass preparation, while the other was subsequently treated with RNAlater (Thermo Fisher Scientific, MA) or flash frozen in liquid nitrogen for gene expression analysis.

### Smeared-slide glass and Giemsa staining of biopsy sample

After fixation, a maximum of 20 mg of fixed gonad fragment was smeared on a slide glass with 12 rounded wells. Each well contained one sample. These slide glasses were stained by Giemsa staining solution for 30 min at room temperature. Giemsa-stained samples on slide glasses were then observed under an optical microscope (Olympus CX21). Sex of Akoya pearl oyster was determined by the presence of sperm or oocytes on the smeared-slide samples.

### Histological analysis of gonad

Gonads of Akoya pearl oyster were collected in the final sampling period in August 2016 (40 months after hatching) and were fixed in Davidson solution. Fixed gonads were embedded in paraffin, cut into 5 μm sections, and stained with Delafield’s hematoxylin–eosin. These gonad sections were observed under a microscope to confirm the sex and gonadal stage.

### RNA extraction and cDNA synthesis

Total RNA extraction from gonad tissue was performed using RNeasy® Plus Universal Mini Kit (QIAGEN, Germany) following the manufacturer’s protocol. The cDNA was synthesized using QuantiTect® Reverse Transcription Kit (QIAGEN, Germany) following the manufacturer’s protocol. Synthesized cDNAs were then diluted 10 times for gene expression analysis using specific primers for genes involved in sex determination.

### Sex-related genes expression analysis

RNA expression of three different sex-related genes, *Dmrt2* (*Double-sex and mab-3-related transcription factor 2*), *Vtg* (*Vitellogenin*), and *Zp* (*Zona pellucida*) in gonad of Akoya pearl oyster were analyzed using reverse transcription polymerase chain reaction (RT-PCR). Two μl of the diluted cDNA solution were used for the PCR reaction (total volume: 25 μl). Amplification procedure of these genes followed protocol of GoTaq® G2 Hot Start Green Master Mix 2X (Promega, USA). The specific primers for each gene were designed using a Blast Search of the *Pinctada fucata* Genome (http://marinegenomics.oist.jp/pearl/blast/search?project_id=36) (Table 1). Amplification was performed with programmable thermal cycler (Biometra Tprofessional BASIC 96 Gradient) consisting of pre-denaturation at 95 °C for 2 min, followed by 40 cycles of denaturation at 95 °C for 15 s, annealing at 55 °C for 15 s, extension at 72 °C for 15 s, and an additional extension step at 72 °C for 5 min. Amplicons were separated on 2% agarose gel (100 V; 15 min) and visualized by staining with ethidium bromide under UV light.

**Table 1:** Nucleotide sequence of primers used in RT-PCR analysis

Primer name		Nucleotide sequence (5′-3′)
*Dmrt2*	Forward primer	ctc cat ttc caa cat tca tac aat a
	Reverse primer	tga tga agt tgc aga ctt tgg t
*Vtg*	Forward primer	gtt atg gag tca gaa ccg ttg a
	Reverse primer	gaa tga agc ggc att tcc
*Zp*	Forward primer	tga agg ttg cca tgg aga gt
	Reverse primer	gat ttg ccc tct aag ttt gat cgt
*GAPDH*	Forward primer	acc act gtc cac gcc att
	Reverse primer	act ctg gta taa ctt tgc cta cgg

## Results

### Sex ratio of Akoya pearl oyster

**Fig. 1:**
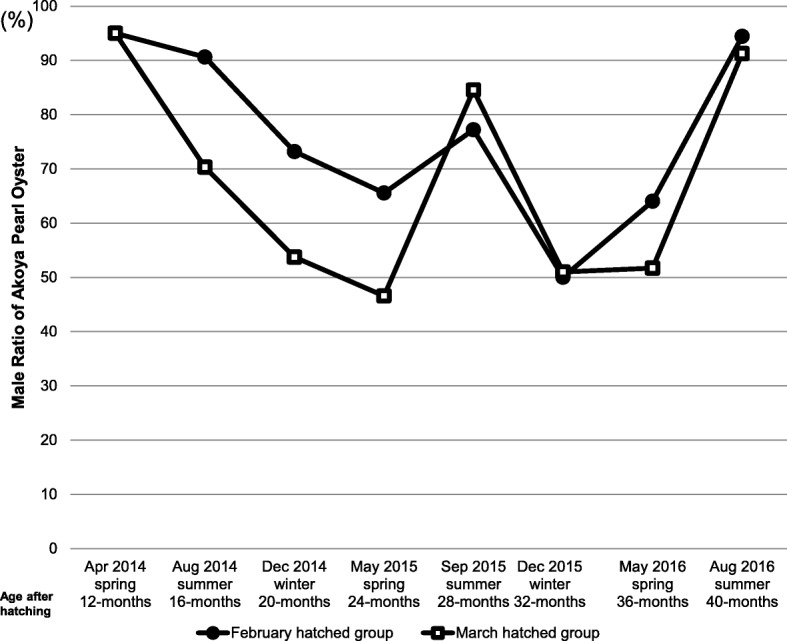
Male ratio of Akoya pearl oyster *(P. fucata)* at seven sampling times. The sex of individual Akoya pearl oysters in two different hatching groups were determined every four months by biopsy. Male ratio was calculated based on the sex obtained from biopsy check

Male ratio based on biopsy results of gonad are shown in Fig. 1. The graph shows fluctuating trends in the male ratio in both groups throughout the study period. As much as 95% of total individuals were males on the first sex check 12 months after hatching. Male ratio always increased from spring to summer, which is the breeding season in Akoya pearl oyster, while it always decreased from summer to winter. Male ratio decreased from the first winter to spring, and then increased in the next such period (Fig. 1).

Of individuals in the total sample, 35% remained male, while only 2% remained female throughout the experimental period, as determined from biopsy results.

### Gonadal stage of Akoya pearl oyster

**Fig. 2:**
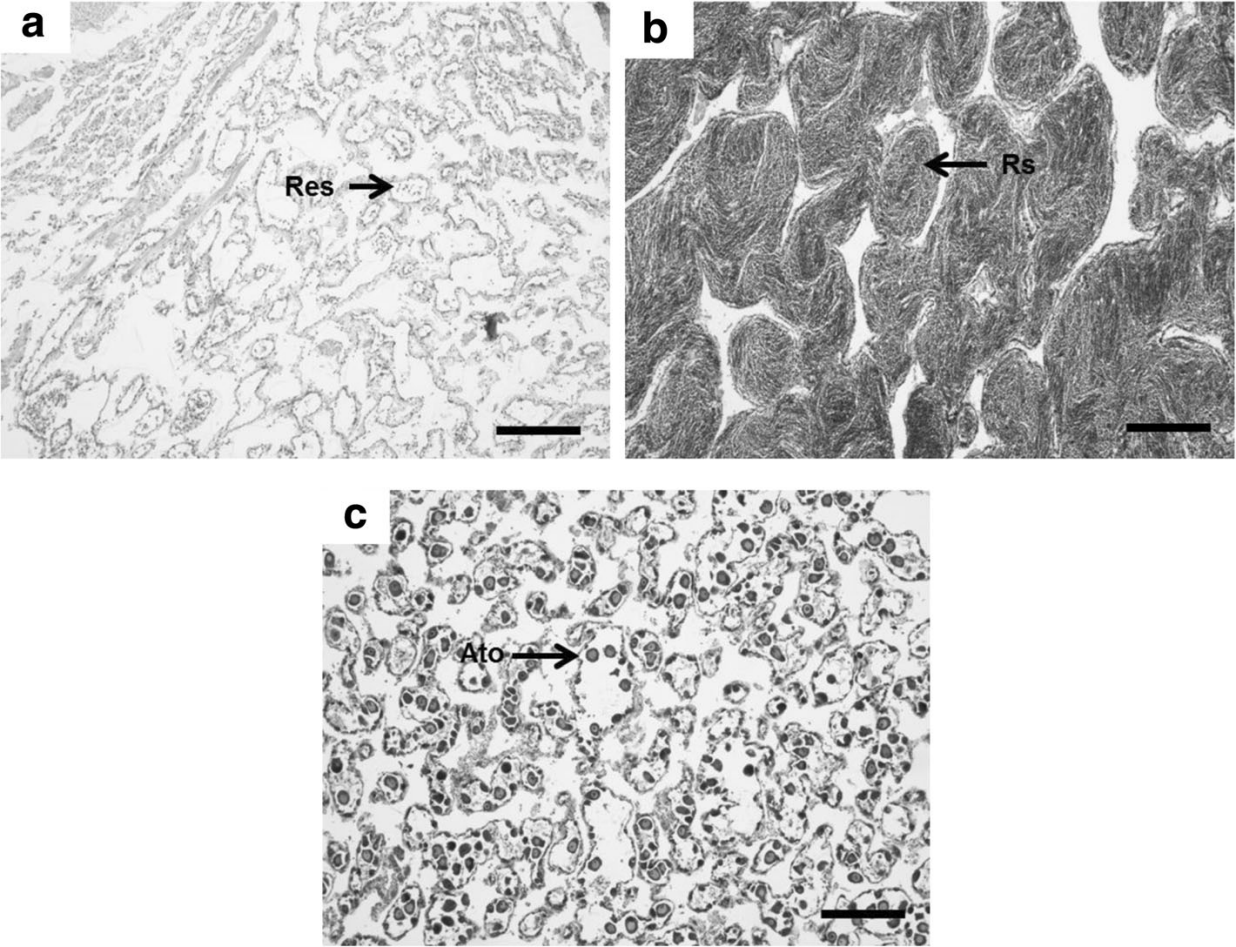
Photomicrographs of transversal section of representative Akoya pearl oyster (*P. fucata*) gonads showing stages in gametogenesis (**a**) male gonad in resting stage with residual spermatozoa, (**b**) male gonad in mature stage with ripe spermatozoa in dense volume, (**c**) female gonad in resting stage with residual or atretic oocytes. Res, residual spermatozoa; Rs, ripe spermatozoa; Ato, atretic oocyte. Scale bar, 200 μm

Gonadal stage was determined by histological observation (Fig. 2). Two types of spermatogenesis stage were observed in male oysters. The mature stage was indicated by the dense volume of ripe spermatozoa, while in the resting or spent stage of spermatogenesis residual spermatozoa filled the acini. All female oysters had already entered the resting stage of oogenesis, as residual oocytes or atretic oocytes were observed in the gonads.

### Determination of sex by biopsy

**Fig. 3:**
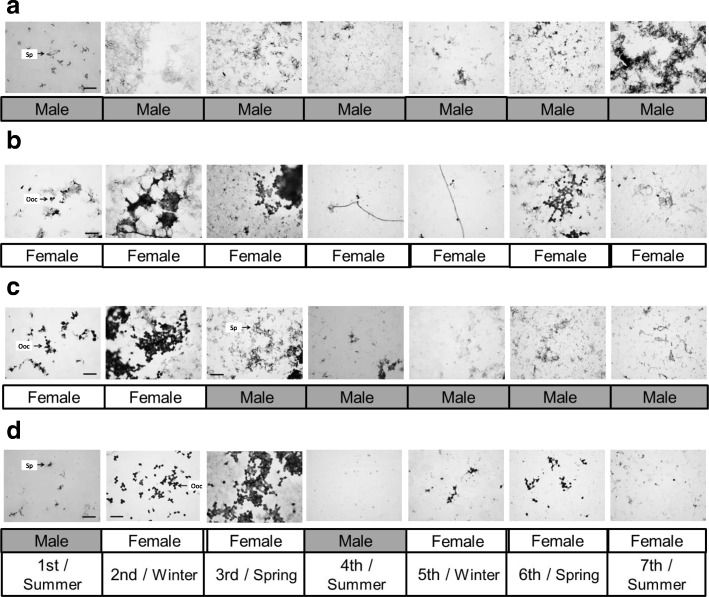
Four typical sex-changing patterns of Akoya pearl oyster (*P. fucata*) (**a**) all-period male, (**b**) all-period female, (**c**) female changed to male, (**d**) changing between male and female. Sex-changing pattern was based from the sex of Akoya checked by biopsy at seven sampling times. Each symbol indicates: Sp, sperms; Ooc, oocyte. Scale bar, 200 μm

There were several typical sex-changing patterns of Akoya pearl oysters found in this experiment, including all-period male (AM) (Fig. 3a), all-period female (AF) (Fig. 3b), female-to-male sex change (FM) (Fig. 3c), and male-to-female sex change (MF2) (Fig. 3d). Sperms were observed in all seven sampling times of all-period male oysters, while oocytes were observed throughout the experimental period in all-period female oysters based on biopsy results.

### Sex-related genes expression

**Fig. 4:**
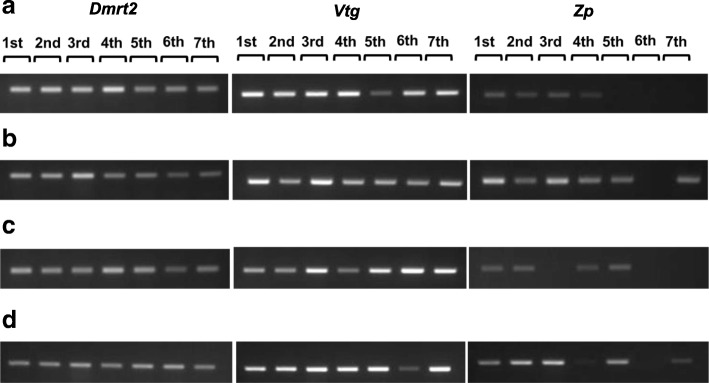
RT-PCR analysis of Akoya pearl oyster (*P. fucata*) *Dmrt2*, *Vtg* and *ZPB* at seven sampling times. (**a**) AM: all-period male; (**b**) AF: all-period female; (**c**) FM: female changed to male; (**d**) MF2: changing between male and female. 1st-7th: sequence of sampling time

RT-PCR analysis showed that *Dmrt2* and *Vtg* were expressed in four representative individuals, which exhibited different sex-changing patterns in all periods. These two genes were expressed in all periods, regardless of the sex. *Zp* was also expressed in both male and female, although not in all periods (Fig. 4). These results indicated that Akoya pearl oyster displays hermaphroditism during its life cycle.

## Discussion

Nearly all one-year old Akoya pearl oysters were males during the first determination of sex in this experiment. Initial functional sex of Akoya pearl oyster is typically male because male germ cells generally mature before female germ cells in [11]. The increase in the number of males during spring to summer is equal to the decrease of the number of females. One of the spawning seasons of Akoya pearl oyster is around June, when the water temperature is warm. Spawning in closely related species of Akoya pearl oyster, such as *Pteria sterna*, *P. imbricata* and *P. radiata*, is initially triggered by rising water temperature [8, 12, 13]. The oocytes are released outside to the water during spawning, so there are only a few or even no oocytes left in the gonad. It is predicted that Akoya pearl oyster always has two kinds of germ cells in a given individual at the same time. Some sperm cells remained in the female gonads after spawning, as spawning is generally incomplete [6]. The sex was determined to be male if only spermatogonia were observed when the gonad was checked by biopsy after the spawning season had passed. Other reasons are that gametogenesis always occurs actively and rapidly [6], and spermatogonia proliferate more rapidly than oogonia [4].

Gonadal stage was observed using transverse sections of Akoya pearl oyster gonads. Whole gonad sampling time was done in August, when the spawning season had already elapsed two months before sampling time. The time of final sampling was the reason why the majority of pearl oysters were in the resting or spent stage of gametogenesis. Other than resting stage of gametogenesis as the majority of gonadal stage, the mature stage was also observed in several individuals. The different gonadal stages observed in *P. fucata* used in this study indicate that this species is a continuous spawner and could be due to continuously active and rapid gametogenesis [6]. Moreover, the reproductive cycle in *P. fucata* is less distinct in tropical temperatures and spawning occurs continuously [14].

The presence of sperm and oocyte in the gonad of Akoya pearl oyster is important because it directly affects the quality and efficiency of cultured pearls [1]. *Dmrt2* (double-sex and mab-3-related transcription factor 2) is an important gene in the maturation of sperm because it plays critical role in spermatocytes and spermatids differentiation into sperms [15]. *Vtg* (vitellogenin) expression suggests the occurrence of vitellogenesis in the form of yolk protein autosynthesis in ovary of marine bivalves [16]. Zona pellucida (ZP) domain proteins are components of the egg envelope in invertebrates [17].

Determination of sex by biopsy revealed that some Akoya pearl oysters possessed oocytes at certain times, and possessed sperm cells without any oocytes at a subsequent biopsy. The same pattern was also observed for individuals with sperm cells at certain sex determination tests, but oocytes were observed in the gonad instead of sperm at the next biopsy. These results indicate that Akoya pearl oyster exhibit phenotypic bidirectional sex-change. However, RT-PCR result of three sex-related genes, i.e. *Dmrt2*, *Vtg* and *Zp* showed that these genes were expressed in all male and female pearl oysters during experimental period. This result was supported by similar gene expression trends of those male- and female-related genes in all types of sex-changing pattern. The concerted expression of three sex-related genes indicates that sperm and oocyte are present at the same time inside the gonad of Akoya pearl oyster. Our results suggest that the Akoya pearl oyster is a simultaneous hermaphrodite species rather than a sex-changing species.

Several previous research studies reported similar results supporting our results in the present study. In addition to being a transitional hermaphrodite, 0.2% of *P. margaritifera* population exhibited simultaneous hermaphroditism [6]. As much as 1.1% of *P. radiata* were simultaneous hermaphroditic pearl oysters, as they had both sperm and oocyte in the gonad concurrently [13]. A few simultaneous hermaphrodites in *P. imbricata* were also observed, although at very low levels [12]. The occurrence of both male and female germ cells in the same gonad was also recorded in *P. albina* [4] and *P. fucata* [18].

In contrast with the findings in this study, *P. margaritifera* develops as male first then progressively changes to female after two years [6, 9]. Another member of genus *Pinctada*, *P. maxima* matures first as male during year one, indicating protandrous hermaphroditism [5]. Similar with *P. margaritifera* and *P. maxima*, *Pteria sterna* was also reported as a potential transitional hermaphrodite [8].

The sex of Akoya pearl oyster was expected to be strongly affected by environmental factors, as females appear at breeding season; however, few individuals with no sex change have been found. These results indicated that sex of Akoya pearl oyster is influenced by both environmental and genetic factors. Studies involving oysters, namely *Crassostrea gigas* [19] and *P. margaritifera* [20], supported the theory that environmental factors combined with genetic mechanism control sex determination of adult oysters. The male and female determining components at certain stages of development are responsive to environmental conditions [11]. Furthermore, the sensitivity of Akoya pearl oyster in response to environmental components must be investigated by whole genome sequencing in order to detect any single nucleotide polymorphisms (SNPs).

## Conclusion

In this study, sex was determined in live oysters individually and continuously for two years, thus yielding a better understanding on the sex characteristics of Akoya pearl oyster. The results of this study suggest for the first time that Akoya pearl oyster is a hermaphrodite with both male and female germ cells in a single individual at the same time. Akoya pearl oyster is a continuous spawner, as indicated by different gonadal stages observed in the same population. Sex determination in adult Akoya pearl oyster may be affected by genetic and environmental factors. These results provide new information concerning relationship between the sex of Akoya pearl oyster and pearl quality. The findings of this study may contribute to high-quality pearl cultivation with higher efficiency.
